# Lack of evidence for cargo release of CD63-EVs into recipient cells

**DOI:** 10.1038/s41598-026-45021-2

**Published:** 2026-03-26

**Authors:** Shaghayegh Askarian-Amiri, Winfried Weissenhorn, Rémy Sadoul, Christine Chatellard

**Affiliations:** University Grenoble Alpes, CEA, CNRS, Institut de BiologieStructurale (IBS), Grenoble, 38000 France

**Keywords:** Endosomes, Membrane fusion

## Abstract

Extracellular vesicles (EVs), including exosomes, are widely believed to mediate intercellular communication by delivering molecular cargo into the cytosol of recipient cells. However, the efficiency and mechanisms of such cargo delivery remain unclear. In this study, we investigated whether EVs bearing the canonical tetraspanin EVs marker CD63 are capable of fusing with recipient cell membranes to release their contents. Using the highly sensitive NanoBiT split luciferase system, we tracked potential fusion events between HiBiT-tagged CD63-EVs and HEK293 cells expressing cytosolic or endosome-localized LgBiT. While HiBiT and LgBiT reconstitution was readily detected when EVs carried the viral fusion protein VSV-G, no luminescence was observed with HiBiT tagged CD63-EVs in absence of VSV-G, despite their uptake or binding by recipient cells. Similarly, EVs carrying HiBiT-HSP70 failed to show evidence of cargo release. These findings demonstrate that unmodified HiBiT-CD63-EVs do not fuse with the plasma or endosomal membranes of recipient cells, suggesting that EV-mediated cargo delivery via membrane fusion is inefficient or absent under standard culture conditions. Our results challenge the prevailing view of EV function in intercellular communication and underscore the need for re-evaluation of EV cargo transfer mechanisms.

**Supplementary Information:**

The online version contains supplementary material available at 10.1038/s41598-026-45021-2.

## Introduction

Cells secrete extracellular vesicles (EVs) either directly from the plasma membrane or through multivesicular endosomes that fuse with the plasma membrane^1^. In this latter case intraluminal vesicles released in the extracellular milieu are referred to as exosomes. EVs allow secretion of biologically active molecules and are thought to allow cell-cell communication. Several studies have suggested that EV cargo can be delivered into recipient cells^2,3^ though the efficiency of this process is generally considered as low^4^. Furthermore, the molecular mechanisms underlying uptake and content delivery of EVs in acceptor cells remain poorly understood. To track exosome secretion and fate in recipient cells, many studies have utilized CD63 fused to GFP^5,2,3^. Indeed, CD63 is a widely accepted EVs marker, highly enriched in the intraluminal vesicles of multivesicular endosomes which give rise to exosomes^1,6^.

CD63-GFP-containing exosomes have been shown to bind to the surface of recipient cells, sometimes followed by endocytosis^7,8^. In one study using HEK293 cells, CD63-labeled exosomes were reported to efficiently enter through endocytic hotspots and traffic within endosomes to sites of contact with the endoplasmic reticulum, where they were hypothesized to fuse and release their cargo^9^. In this study, we aimed to demonstrate the potential fusion of CD63-bearing EVs released by HEK293 cells with HEK293 recipient cell membranes using the luminescence NanoBiT system.

**Fig. 1:**
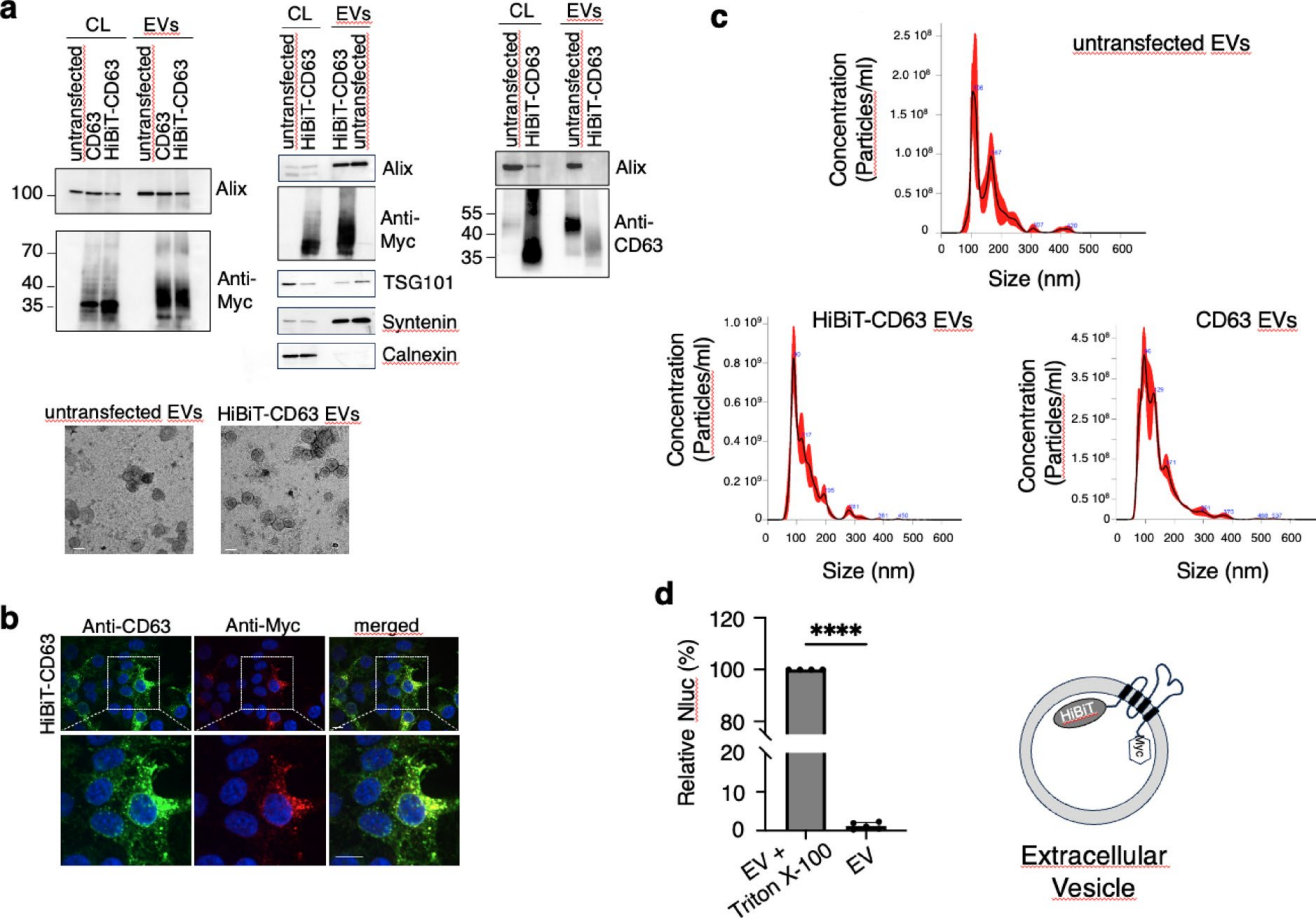
HiBiT-CD63 EV characterization. (**a**) Characterization of EVs secreted by HEK293 cells expressing HiBiT-CD63-Myc. (left panel) Western blot analysis of cell lysates (CL, 5 µg proteins) and EVs (1 µg; 4 × 10^9^ particles). CD63 and HiBiT-CD63 were revealed using anti-myc polyclonal antibody, while Alix was revealed using an anti-Alix polyclonal antibody. (middle panel). Western blot comparison of EVs and cell lysates from untransfected and HiBiT-CD63 transfected cells. Anti-myc was used to reveal the presence of HiBiT-CD63, while antibodies against Alix, syntenin and calnexin were used to characterize the presence of the endogenous proteins. (right panel) Western blot analysis performed under non-reducing condition to demonstrate the presence of CD63 and HiBiT-CD63-Myc. For untransfected cells, 10-fold higher amounts of proteins and particles were loaded compared to HiBiT-CD63 transfected samples. CD63 was revealed using anti-CD63 monoclonal antibody and Alix was revealed using anti-Alix polyclonal antibody. (bottom panel) TEM images of EVs isolated from untransfected or HiBiT-CD63 transfected cells. Scale bar, 80 nm. (**b**) HiBiT-CD63-Myc is located on vesicular structures, similarly to endogeneous CD63. Maximum intensity of confocal microscopy images of HEK293 cells transfected with HiBiT-CD63. Cells were stained with an anti-CD63 monoclonal antibody (green) or anti-myc polyclonal antibody (red). Scale bar, 10 μm. (**c**) The size and concentration distribution of EVs isolated from transfected cells do not differ from non-transfected cells as determined by NTA. The black line represents mean values of 3 videos, and the red shade the standard deviation. (**d**) Left: EVs were incubated with the cytosol of LgBiT expressing cells, with or without 0.5% of TritonX-100. Luminescence of detergent solubilized EVs was set to 100%. Mean +/- SD of four independent wells from five experiments. Student’s t test paired, two-tailed (****p < 0.0001). The diagram on the right explains the theoretical orientation of HiBiT-CD63-Myc with the 11-amino-acid peptide, HiBiT and the Myc tag fused to the N-terminal and C-terminal region of CD63, respectively. Both tags are located within the EV lumen.

The NanoBiT system is based on split luciferase complementation between two NanoLuc fragment: an 18-kDa polypeptide Large BiT (LgBiT), and an 11-amino-acid peptide, HiBiT. HiBiT binds to LgBiT with high affinity (Kd = 0.7 nM), thereby forming a stable complex that reconstitutes active NanoLuc luciferase.This system has previously been used to monitor the entry and release of HIV and virus-like particles containing HiBiT in cells expressing LgBiT^10,11^. In our approach, we incubated EVs purified from cells expressing HiBiT fused to the N-terminal region of CD63, which is localized within the EV lumen (Fig. 1d), with cells expressing cytoplasmic LgBiT. Fusion of EVs with recipient cells should enable interaction between cytosolic LgBiT and HiBiT-CD63 thereby increasing luminescence. We found that such a luminescence increase did not occur with HiBiT-CD63 EVs and could only be detected with vesicles containing also the viral fusion protein VSV-G. The absence of detectable luminescence observed with HiBiT-CD63 EVs indicates that fusion of CD63-containing-EVs with receiving cells does not occur.

## Materials and methods

### Cells culture and plasmids

The human embryonic kidney cell line (HEK293T, ATCC CRL-3216) was maintained in Dulbecco’s Modified Eagle Medium (DMEM) supplemented with 10% heat-inactivated fetal bovine serum (FBS) (Gibco). HEK293 cells were incubated at 37 °C in a humidified atmosphere with 5% CO_2_. All cell lines were routinely tested for mycoplasma contamination using MycoAlert Mycoplasma Detection Kit (Lonza).

To generate the HiBiT-CD63-myc construct, HiBiT-Linker primers (Table 1) were paired and inserted into the hCD63-myc pcDNA3.1 vector using the BamH1 restriction site. GFP-CD63 construct was a generous gift from J. Gruenberg’s laboratory (Geneva, Switzerland). HSP70 cDNA was obtained from mouse brain cDNA via RT-PCR, using a forward primer incorporating the HiBiT sequence and a reverse primer containing the myc sequence (Table 1). The resulting HiBiT-HSP70-myc cDNA was inserted into the Nhe1/Xba1 sites of the LgBiT expression vector (pCMV-LgBiT, Promega), to remove LgBiT cDNA and generating the pCMV-HiBiT-mHSP70-myc plasmid. LgBiT-hRAB5 and LgBiT-dRAB7were constructed by amplifying (Table 1) and inserting hRAB5 or dRAB7 cDNA at the C-terminus of LgBiT into the BamH1 site of the pCMV-LgBiT vector. mRFP-hRAB5 and mRFP-dRAB7 and pCMV-VSV-G plasmids were purchased from Addgene (#14437, #14436 and #8454 respectively). All plasmids were transformed into Top10 *E. coli* and subsequently purified using the Endotoxin-Free Plasmid Midiprep Kit (Macherey-Nalgene).

### Transfection

Transient transfection of HEK293 cells was performed using the calcium phosphate method. Briefly, 24 h prior to transfection, cells were seeded at a density of 4 × 10⁴ cells/cm² on poly-ornithine-coated plates in culture medium. For co-transfections, the DNA ratio was 1:4 for VSV-G to HiBiT-CD63. Plasmids encoding the proteins of interest were diluted in sterile water and a CaCl_2_ solution (0.26 M final concentration), then mixed with an equal volume of 2× HEPES Buffered Saline (Sigma-Aldrich), gently bubbled to mix, and incubated for 20 min. The resulting DNA solution was added dropwise to the plated cells. Six hours later, the medium was aspirated and replaced with fresh culture medium.

### EVs isolation

Extracellular vesicles (EVs) from HEK293 cells were purified by differential centrifugation of cell culture supernatants. Briefly, HEK293 cells were transfected, and 6 h later, the cells were washed twice with PBS and incubated for 40 h in exosome-free medium (DMEM with 10% FBS, ultracentrifuged at 140 000 g for 18 h). The conditioned medium from the HEK293 cells was then subjected to serial centrifugation to remove cells, debris, and large vesicles: first at 2 000 g for 10 min, followed by 20 000 g for 30 min, collecting the supernatant and discarding the pellets. The medium was subsequently passed through a 0.22 μm sterile filter (Millex GV PVDF, Millipore). EVs were collected by ultracentrifugation at 100 000 g for 1.5 h and washed in filtered PBS before undergoing a second centrifugation at 100 000 g for 1.5 h. The final EVs were resuspended in PBS or Opti-MEM and immediately used for NTA or applied to the receiving cells.

### Western blot analysis of cell lysates and EVs

Cell lysates were collected simultaneously with the EV supernatants. Briefly, cell lysis was performed using a buffer containing 20 mM Tris-HCl (pH 7.5), 150 mM NaCl, 1 mM MgCl_2_, 1% Triton X-100, and a complete, EDTA-free protease inhibitor cocktail (Roche). The lysates were clarified by centrifugation at 16 000 g for 5 min at 4 °C, and the supernatant was transferred to fresh tubes and frozen at -20 °C. Protein concentration was determined using the bicinchoninic acid (BCA) assay (Pierce). For each sample, cell lysate (CL) or particles were separated on SDS-polyacrylamide gels, and the proteins transferred to PVDF membranes (Millipore). The membranes were blocked in TBS-T milk solution (TBS, 0.05% Tween 20, 5% non-fat milk). Primary antibodies used included anti-myc rabbit polyclonal antibody (1:2000, Proteintech), anti-Alix rabbit polyclonal antibody (1:1000, Covalab), anti-Calnexin rabbit polyclonal antibody (1:1000, StressGen), anti-Syntenin rabbit polyclonal antibody (1:1000, Abcam), anti-VSV-G rabbit polyclonal antibody (1:2000, Sigma-Aldrich), anti-CD63 mouse monoclonal antibody (1:250, clone Ts63, Invitrogen), anti-LgBiT mouse monoclonal antibody (1:1000, Promega), anti-TSG101 rabbit monoclonal antibody (1:1000, clone E6V1X, Cell Signaling), anti-Tubulin mouse monoclonal antibody (1:1000, clone α3A1, generous gift from A. Andrieux’s laboratory). The membranes were then incubated with anti-rabbit IgG or anti-mouse IgG conjugated to HRP (1:10 000, Jackson ImmunoResearch) in TBS-T-milk solution for 1 h at room temperature. Immunoreactive bands were visualized using a solution containing 100 mM Tris-HCl (pH 8.5), 1.25 mM luminol, 2 mM p-coumaric acid, and 0.009% H_2_O_2_, and detected using the ChemiDoc MP Imager system (Biorad).

### Immunofluorescence

Immunofluorescence was performed after fixing cells grown on glass coverslipsfor 20 min with 4% PFA in PBS. All steps of the immunofluorescence procedure were performed at roomtemperature. After fixation, cells were permeabilized and blocked in PBS containing 0.5% Triton X-100 and 5% Goat Pre-Immune serum (GPI) for30 min, followed by 1 to 2 h incubation with the primary antibodies diluted in blocking solution. After washing primary antibodies with PBS, the cells were incubated for 1 h with secondary antibodies conjugated to Alexa Fluor 488 or Alexa Fluor 594 (Invitrogen) diluted in blocking solution, followed by PBS washes. Nuclei were stained using Hoechst 33,258 (Sigma). Coverslips were mounted in Mowiol (Calbiochem)and cells were imaged with a spinning disk confocal microscope (Olympus) using a 60xor a 100x oil objective (Olympus) and Metamorphimaging software.

### Post nuclear supernatant of cells expressing LgBiT

Cells expressing LgBiT were washed two times with PBS, incubated within 20 mM Tris-HCl (pH 7.5), 150 mM NaCl, 1 mM MgCl_2_ and gently detached from the plate using a scrapper. Cells were disrupted by sonication and lysate was centrifuged for 10 min at 1500 g. The supernatant was then ultracentrifuged at 100 000 g for 1 h and the supernatant was recovered as the cytosolic fraction.

### Nanoparticle tracking analysis (NTA)

The size and concentration distribution of EVs were measured using a NanoSight NS300 equipped with a 488 nm laser (Malvern Panalytical, Malvern, United Kingdom). EV samples were diluted 10- to 1 000-fold in filtered PBS to achieve a recording of 10–100 particles per frame. The movement of EVs was captured in three separate 60-second videos at camera level 15, and analyzed using NTA3.2 software (Malvern Panalytical). The data presented were the averages obtained from the analysis of three 60 s videos for each sample.

### Electron microscopy

EVs samples were visualized by negative-stain transmission electron microscopy (TEM) using 4 µL of EVs solution (5 × 10^8^particles). Samples were applied for 10 s onto a mica carbon film and transferred to 400-mesh Cu grids that had been glow discharged at 20 mA for 30 s and then negatively stained with 2% uranyl acetate for 20 s. Data were collected on a FEI Tecnai T12 LaB6-EM operating at 120 kV accelerating voltage using a TVIPS F416 camera.

### HiBiT- EVs uptake

Receiving HEK293 cells were transfected with plasmids encoding LgBiT proteins one day after plating. Six hours post-transfection, cells were trypsinized and seeded at a density of 1.5 × 10⁴ cells/well into poly-ornithine-coated white flat-bottom 96-well plates (ThermoScientific), then incubated for 24 h. Fresh EVs purified from cells expressing a HiBiT-fused protein were added to the receiving cells at 2 × 10⁹ particles per well and incubated at 37 °C for the specified time period. At the indicated time, cells were washed twice with PBS and incubated with Opti-MEM, and when indicated, with 0.1 µM DrkBiT for 10 min at 37 °C. Nano-Glo substrate (Nano-Glo Live Cell Assay System; Promega) was then added according to the manufacturer’s instructions and incubated for 20 min at 37 °C. In some experiments, EVs were incubated in the presence of Nano-Glo substrate. Luminescence activity was measured using a ClarioStar microplate reader (BMG LabTech).After luminescence reading, 0.01% digitonin or 0.5% TritonX-100 were added to the same well, incubated 15 min at 37 °C. Luminescence activity was measured under the same condition as above.

### GFP-EVs uptake

HEK293 cells were seeded 24 h before the uptake experiment on poly-ornithine coated glass coverslips. Cells were incubated with GFP-CD63 EVs or GFP-CD63 + VSV-G EVs (6 × 10^9^ EVs/5 × 10⁴ cells) for 6 h at 37°C. Then cells were washed three time with PBS, fixed with 4% PFA for 20 min and washed with PBS. Nuclei were stained using Hoechst 33258 (Sigma). Coverslips were mounted in Mowiol (Calbiochem) and cells were imaged with a spinning disk confocal microscope (Olympus) using a 60x objective (Olympus) and Metamorph imaging software. Stack image files were processed with Imaris10.2.0 software (Bitplane) for cell segmentation and vesicle detection. Cells were segmented in 3D using the ‘Surface’ modulewith machine learning-based segmentation. Vesicles were subsequently identified within the segmented cell volumes using the ‘Spots’ module, which detects spherical structures based on size and intensity thresholds. The quantification of intracellular vesicles was performed with split spot into surface object method. This approach enabled precise localization and quantification of intracellular vesicles in three dimensions.

### Single vesicle imaging

EVs from HiBiT-CD63 /VSV-G double-transfected HEK293 cells were spotted onto coverslips previously ionized by plasma technology and left bound for 20 min. The EVs were incubated 20 min in 4% PFA solution, washed 3 times with PBS and then saturated using 5% BSA and 5% goat pre-immune serum. Immunostaining was performed using anti-CD63 monoclonal antibody (clone H5C6, BD Pharmigen) and anti-VSV-G rabbit polyclonal antibody andthen revealed with anti-mouse IgG Alexa488 and anti-rabbit IgG Alexa594 secondary antibodies (Invitrogen). The coverslips were washed and mounted in Mowiol (Calbiochem) for observation using a 100 X oil objective of an IX83 inverted microscope (Olympus).

### Data analysis/statistical analysis

**Fig. 2:**
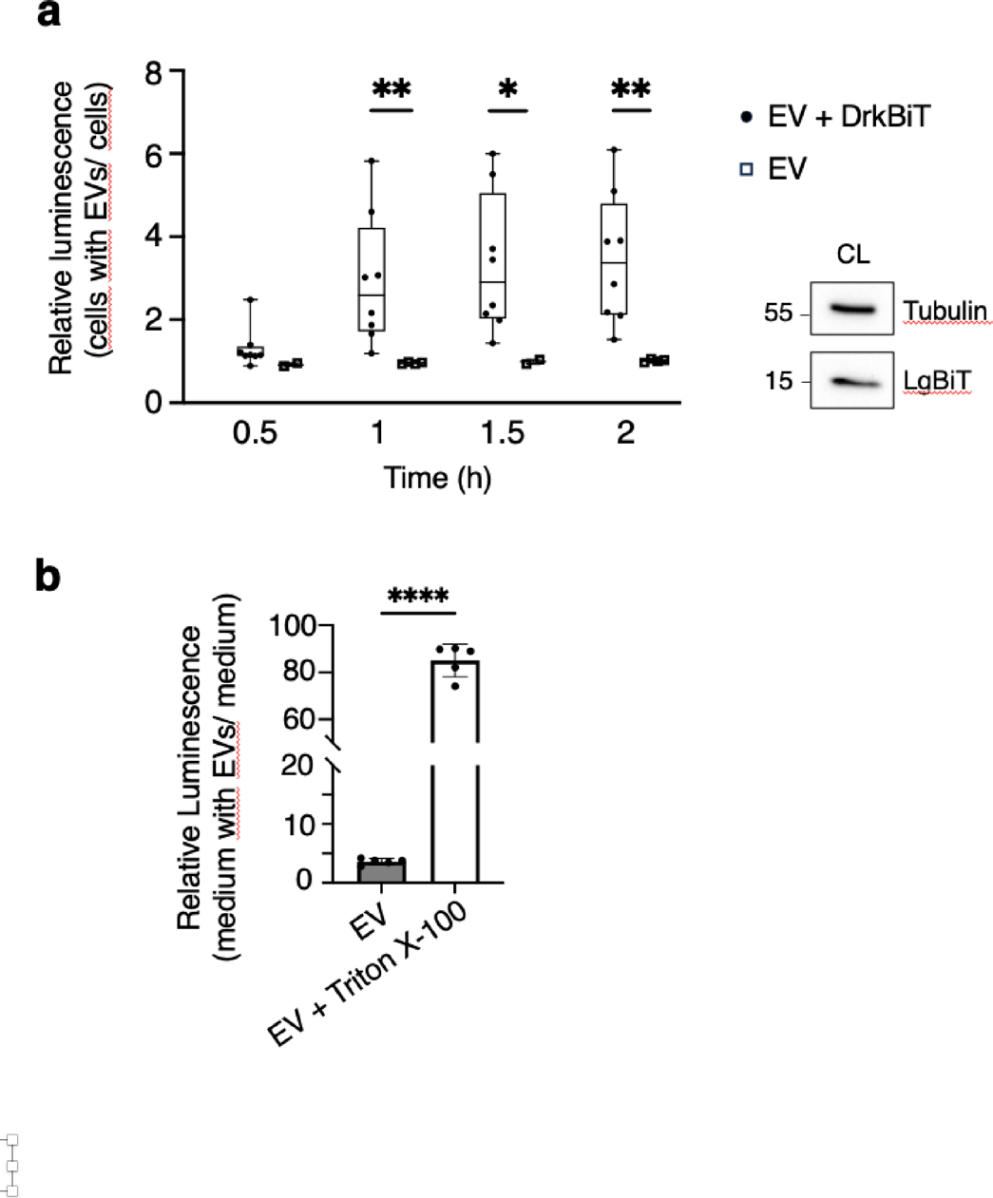
Luminescence increase over time is caused by LgBiT from the culture medium of LgBiT cells and is blocked by DktBiT. (**a**) HiBiT-CD63 EVs were incubated on LgBiT-expressing-HEK293 cells together with the Nano-Glo substrate and 0.1µM of DrkBiT peptide. Luminescence was measured 0,5 h, 1 h, 1,5 h and 2 h later. Median (q1; q3), *n* = 8 independent experiments for EVs and *n* = 4 independent experiments for EVs+ DrkBit. Statistically significant differences were calculated using Mann-Whitney’s U test (*p < 0.05, **p < 0.01). insets: WB analysis with anti-LgBiT antibody show expression of LgBiT in receiving cells. (**b**) Luminescence was measured in cleared medium of LgBiT expressing cells added of HiBiT-CD63 EVs incubated in absence or presence of 0.5% of TritonX-100. Mean +/- SD of five independent wells from one representative experiment. Statistically significant differences were calculated using Student’s t test paired, two-tailed (****p < 0.0001).

Statistical analysis was performed using GraphPad Prism 10 (GraphPad Software, San Diego, CA) with statistical significance considered at *p* ≤ 0.05. Statistical significance was assessed for non-parametric data by Kruskal-Wallis’s test with a post-hoc Dunn’s multiple comparisons test (unpaired, two-tailed) (Fig. 5a), Mann-Whitney’s U test (unpaired, two-tailed) (Fig. 2a). For parametric data, normality of data was assessed using Shapiro-Wilk test then statistical significance was assessed by Student’s t test (paired, one-tailed) (Figs. 1d and 2b).

**Table 1:** Primers used to generate plasmids.

HiBiT-CD63-myc	Forward	5’-TACCGAGCTCGGATCCATGGTGAGCGGCTGGCGGCTGTTCAAGAAGATT AGCGGGAGTTCTGGCGGCTCGAGCGGTGGATCCATGGCGGTGG-3’
	Reverse	5’-CCACCGCCATGGATCCACCGCTCGAGCCGCCAGAACTCCCGCTAATCTT CTTGAACAGCCGCCAGCCGCTCACCATGGATCCGAGCTCGGTA-3’
HiBiT-HSP70-myc	Forward	5’-TCACTATAGGGCTAGATGGTGAGCGGCTGGCGGCTGTTCAAGAAGATTAG CGGGAGTTCTGGCGGCTCGAGCGGTGGATCCGCCAAGAACACGGCGATC-3’
	Reverse	5’-CCTAGGTGTTTCTAGCTACAGATCTTCTTCAGAAATAAGTTTTTGTTCATCCA CCTCCTCGATGGTGGGTCCT-3’
LgBiT-RAB5	Forward	5’-CATCAACAGTGGATCGTCCGGACTCAGATCTCGAG-3’
	Reverse	5’-CCTAGGTGTTGGATCTTAGTTACTACAACACTGATTCCTGG-3’
LgBiT-RAB7	Forward	5’-CATCAACAGTGGATCCCTGTACAAGTACTCAGATCTCGAG-3’
	Reverse	5’-CCTAGGTGTTGGATCTTAACAACTGCAGCTTTCTGCG-3’

## Results

### Characterization of EVs containing HiBiT-CD63

Extracellular vesicles (EVs) secreted by HEK293 cells expressing either HiBiT-CD63-myc or CD63-myc were isolated using ultracentrifugation. Western blot analysis using anti-Myc antibody shows a significant enrichment of both HiBiT-CD63-myc and CD63-myc in the isolated EVs even though HiBiT-CD63-Myc was incorporated into EVs at a slightly lower level (1.2x less) than CD63-Myc (Fig. 1a, left). As expected, WB characterization of EVs secreted by HEK293 cells revealed the strong enrichment of Alix and syntenin, presence of TSG101 and absence of calnexin (Fig. 1a middle). This expression pattern was unchanged in EVs isolated from HiBiT-CD63-myc -expressing cells. WB analysis with an anti-CD63 antibody performed on both cell lysates and EVs derived from untransfected cells and HiBiT-CD63-Myc–expressing cells detected endogenous CD63 as well as HiBiT-CD63. As expected, HiBiT-CD63 was strongly overrepresented relative to endogenous CD63 (Fig. 1a right). Consistent with these findings, HiBiT-CD63-myc detected by anti-Myc in transfected cells was present on vesicular structures closely resembling the distribution pattern of those decorated by CD63 in untransfected cells (Fig. 1b). Furthermore, EM characterization of both preparations showed no obvious morphological changes due to the presence of HiBiT-CD-63-myc (Fig. 1a, EM photographs). NTA analysis demonstrated no notable alteration in the size of EVs secreted by HiBiT-CD63 cells compared to control (Fig. 1c). However, in line with previous findings by Sung et al. (2020)^12^, CD63 overexpression appeared to increase the number of the smallest secreted EVs.

To verify the correct orientation of CD63 and the integrity of EVs purified by ultracentrifugation, we incubated HiBiT-CD63 EVs with LgBiT contained in cytosolic fractions prepared from LgBiT-expressing cells. After substrate addition, the recorded luminescence was less than 2% of that measured in the presence of TritonX-100, indicating that over 98% of HiBiT is enclosed within the EVs (Fig. 1d). This percentage, which is likely even higher as TritonX-100 reduces NanoLuc-induced luminescence^13^ shows that the vast majority of HiBiT fused to the N-terminal part of CD63 points inside EVs suggesting a correct orientation of the protein in the vesicles. Interaction of LgBiT with HiBiT in untreated EVs might come from a flipped orientation of CD63 or from EVs damaged during the isolation procedure.

### Incubation of EVs containing HiBiT-CD63 on LgBiT-expressing HEK293 cells does not increase luminescence

We then incubated HiBiT-CD63 EVs with LgBiT-expressing HEK293 cells. In the initial set of experiments, cells were incubated with EVs together with the cell-permeant luciferase substrate and measurements made after 0.5 h, 1 h, 1.5 h and 2 h. Luminescence increased progressively with incubation time (Fig. 2a). However, no increase was detected when the Nano-Glo substrate was applied in the presence of the DrkBiT peptide, a HiBiT variant with a single Arg-to-Ala substitution that binds to LgBiT but does not restore NanoLuc activity (Fig. 2a). DrkBiT being membrane impermeable^11^, the luminescence detected in absence of DrkBiT is likely to reflect EVs with HiBiT exposed on the outside interacting with LgBiT released by receiving cells during incubation with the Nano-Glo substrate. Accordingly, luminescence was detected upon incubation of HiBiT-CD63 EVs with centrifuged culture media of LgBiT-expressing cells (Fig. 2b), suggesting leakage of LgBiT from receiving cells. As expected, TritonX-100 lysis of the EVs, which exposed the luminal HiBiT, resulted in a several-fold increase in luminescence. The increase in luminescence correlating with the incubation time of vesicles on cells therefore likely reflects the increase in LgBiT released by cells during the incubation with EVs.

**Fig. 3:**
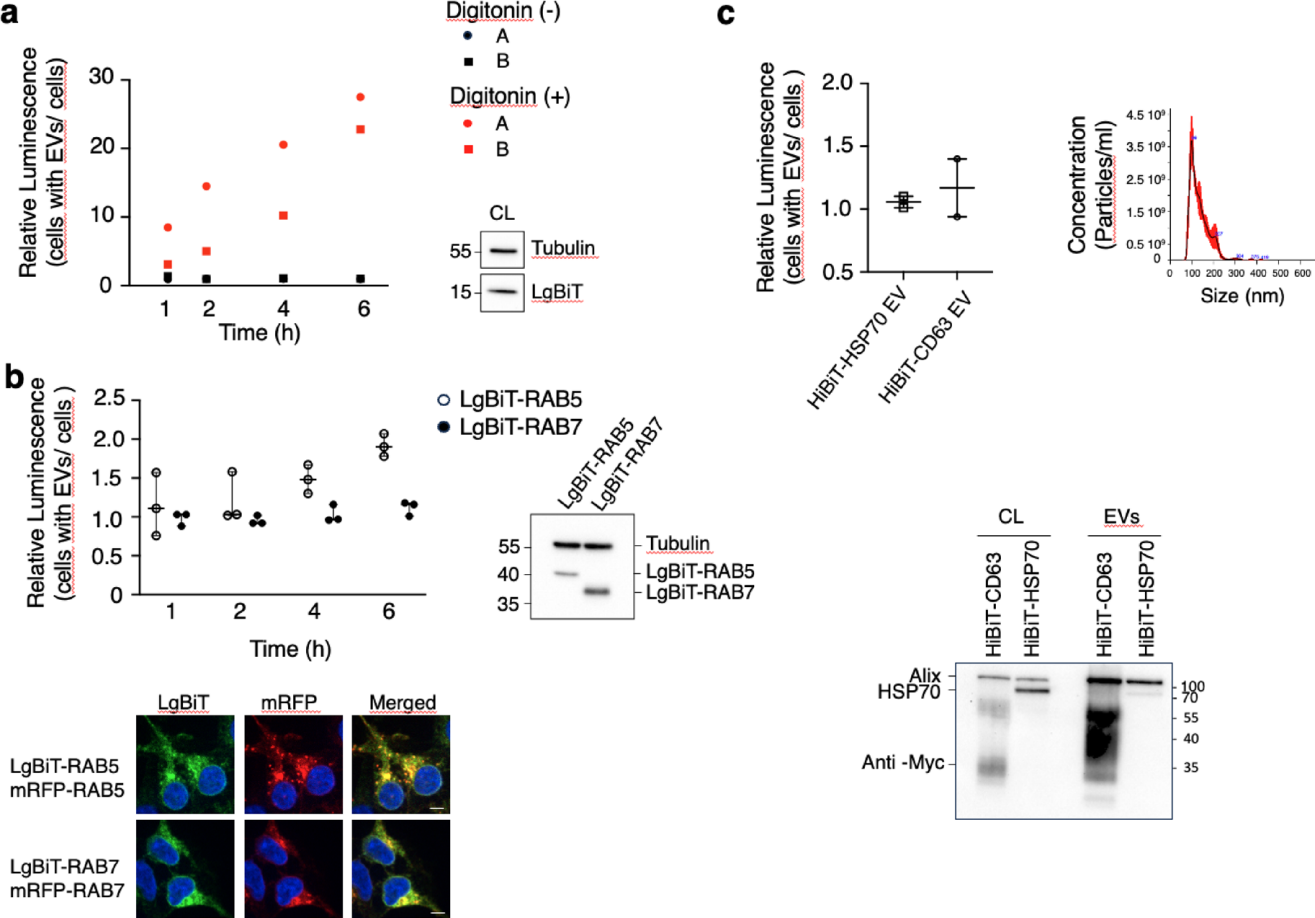
Binding and uptake of HiBiT-CD63 EVs by HEK293 cells. (**a**) HiBiT-CD63 EVs were incubated with LgBiT expressing HEK293 cells. At the indicated times, cells were washed and luminescence activity measured 30 min after addition of DrkBiT and Nano-Glo substrate. Relative luminescence values from two independent experiments (A and B) are shown before (black symbols) and after (red symbols) addition of digitonin to induce LgBiT leakage from cells and access to HiBiT contained in the lumen of EVs. Data represent the mean of three technical replicates from two independent experiments. Expression of LgBiT protein in receiving cells was revealed by anti-LgBiT antibody. (**b**) Incubation of EVs on cells expressing LgBiT-RAB5, or -RAB7 does not indicate fusion of HiBiT-CD63 EVs with endosomal membranes. HiBiT-CD63 EVs were incubated on LgBiT-RAB5 -expressing HEK293 cells (white dots) or LgBiT-RAB7 expressing HEK293 cells (black dots). Median (q1; q3) n= three independent experiments. Photographs of receiving cells show that LgBiT-RAB proteins predominantly localize to vesicular structures, consistent with the known distribution of mRFP-RAB5 or -RAB7expression. Confocal microscopy images of HEK293 cells co-transfected with LgBiT-RAB5 and mRFP-RAB5 or LgBiT-RAB7 and mRFP-RAB7 immunostained with an anti-LgBiT monoclonal antibody. Scale bar, 5 μm. WB analysis using anti-LgBiT demonstrates expression of LgBiT-RAB5 and LgBiT-RAB7 in receiving cells. (**c**) Upper left panel: HiBiT-HSP70 containing EVs do not fuse with LgBiT expressing cells. Luminescence activity of LgBiT-expressing cells was measured after a 3 h incubation of EVs containing HiBiT-HSP70 or HiBiT-CD63 with LgBiT-expressing cells. The DrkBiT peptide was added 10 min before the Nano-Glo substrate. Median (q1; q3) of five wells from two experiments. Upper right panel: The size and concentration distribution of EVs isolated from transfected cells do not differ from non-transfected cells as determined by NTA. The black line represents mean values of three videos, and the red shade the standard deviation. Lower panel: HiBiT-HSP70 is loaded in EVs secreted by transfected HEK293 cells. 10 µg of protein cell lysate and 1 µg of EV proteins (4 × 10^9^ particles) were run on SDS-PAGE and blotted. HiBiT-CD63 and HiBiT-HSP70 were revealed using anti-myc polyclonal antibody, while Alix was revealed using an anti-Alix polyclonal antibody.

Next, LgBiT-HEK293 cells were incubated with HiBiT-CD63 EVs for increasing time periods, alongside a low amount of DrkBiT. Unbound EVs were washed away and luminescence detected after addition of the Nano-Glo substrate. As shown in Fig. 3a, there was no time-dependent increase in luminescence even after 6 h incubation. However, we could detect some luminescence when digitonin was added before the measurement to permeabilize both EVs and cells. This was particularly true for longer incubation times reflecting a time increase in the number of EVs in the wells. The fact that luminescence is only seen upon digitonin permeabilization suggests that EVs bind to the cell substrate or to cells and are possibly endocytosed by LgBiT-expressing cells, but that no membrane fusion required for the interaction between LgBiT and HiBiT, has occurred, even after 6 h incubation of EVs on cells.

All of the above experiments were conducted with LgBiT expressed in the cytosol of recipient cells. Since EVs have been suggested to fuse with endosomes^9^, we next explored the use of LgBiT fused to proteins located on the cytosolic surface of endosomes. HiBiT-CD63 EVs were incubated with HEK293 cells expressing LgBiT fused to either RAB5 or RAB7 (Fig. 3b), proteins associated with early and late endosomes, respectively. As shown by immunostaining of doubly transfected cells, LgBiT fusion to the N-terminus of RAB5 or RAB7 does not alter the localization of these proteins, which colocalize on endosomes with mRFP-RAB5 and RAB7 (Fig. 3b). However, even in this case no fusion of CD63-bearing EVs with endosomal membranes could be demonstrated since luminescence remained near background levels at all times tested.

**Fig. 4:**
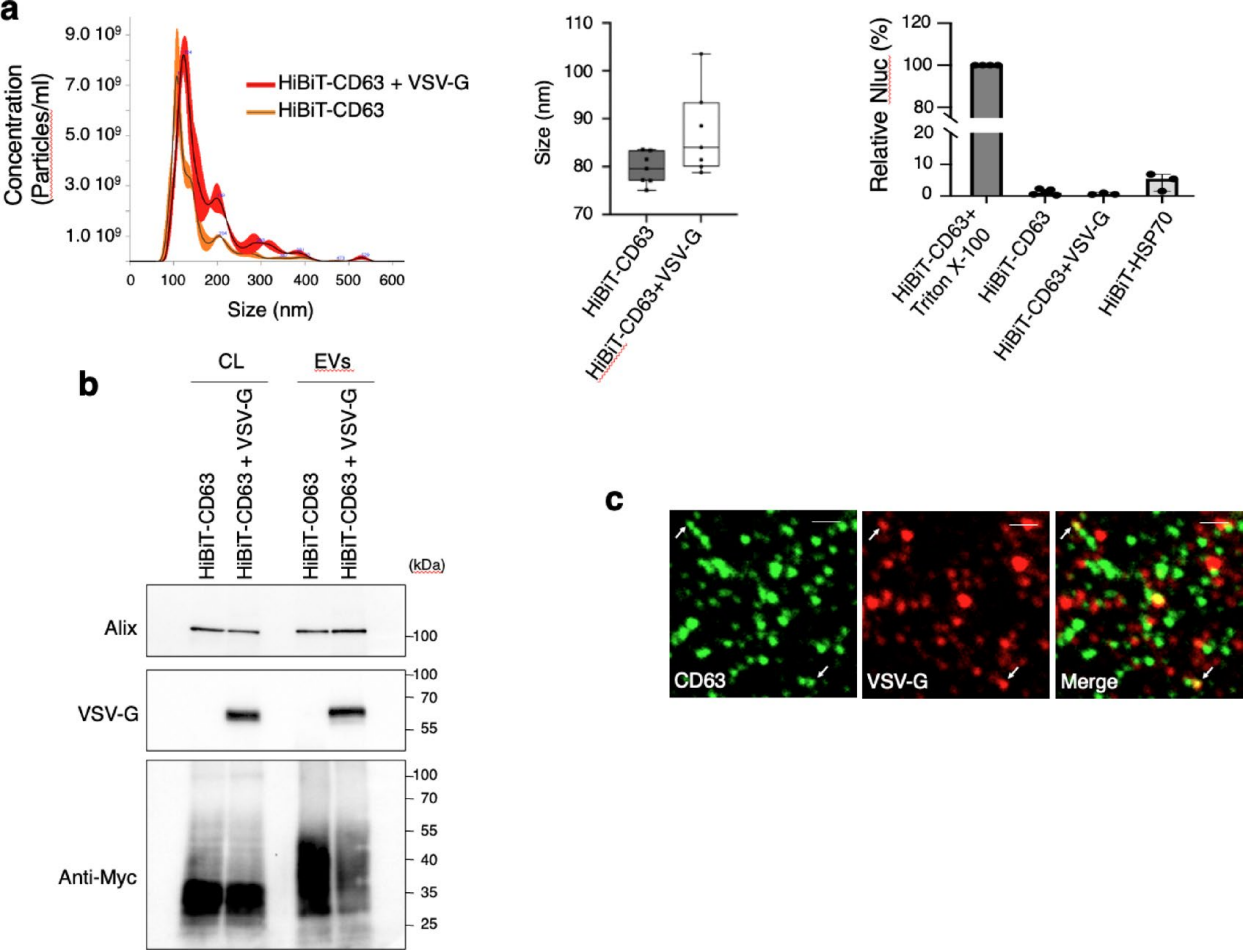
Characterizationof EV containing HiBiT-CD63 and VSV-G proteins. (**a**) Left panel: NTA analysis of HiBiT-CD63 EVs and HiBiT-CD63 + VSV-G EVs. The black line represents mean values of three videos and the red shade represents the standard deviation. Middle panel: The particle size from NTA was measured and compared between both groups. Median (q1; q3) of seven independent experiments. Right panel: EVs were incubated with the cytosol of LgBiT expressing cells, with or without 0.5% of TritonX-100. Luminescence of detergent solubilized EVs was set to 100%. The results shown for HiBiT-CD63 EVs are the same as in Fig. 1d and placed on this graph solely for comparison with HiBiT-CD63 EVs secreted by cells expressing VSV-G and HiBiT-HSP70 EVs. Mean +/- SD of four independent wells from three experiments. (**b**) VSV-G is incorporated in EVs purified from cells expressing both HiBiT-CD63 and VSV-G. Western blot analysis of lysates and EVs secreted by cells transfected with HiBiT-CD63 or HiBiT-CD63 together with VSV-G. HiBiT-CD63 were revealed using anti-myc polyclonal antibody, while VSV-G and Alix were revealed using an anti-VSV-G polyclonal antibody and an anti-Alix polyclonal antibody, respectively. (**c**) VSV-G protein can be detected in some EVs containing CD63. EVs purified from HiBiT-CD63/VSV-G double-transfected HEK293 cells were spotted onto coverslips, stained with anti-CD63 monoclonal (green) and anti-VSV-G polyclonal (red) antibodies, and then imaged using confocal fluorescence microscopy. Arrows indicate double-labelled EVs. Scale bar = 1 μm.

Finally, we also used HiBiT fused to HSP70, which has been shown to concentrate inside EVs^14,3^ to monitor cytosolic release of EV cargoes. Western blot analysis showed that HSP70 is loaded even if not enriched in EVs (Fig. 3c). NTA analysis of HiBiT-HSP70 EVs showed that cells transfected with HSP70 secrete EVs with concentration and size distribution similar to those of CD63 EVs (Fig. 3c). Most of HiBiT-HSP70 (94%) was present inside EVs as revealed by the luminescence in presence of LgBiT before and after TritonX-100 EVsolubilization. (Fig. 4a right panel).

Futhermore, as in the case of HiBiT-CD63 EVs, no sign of increase in luminescence could be detected upon incubation of HiBiT-HSP70 containing EVs with LgBiT-expressing cells (Fig. 3c).

Thus, our results suggest that despite EVs binding and possibly endocytosis by recipient cells, EVs do not fuse with membranes of the receiving cells, as evidenced by the lack of HiBiT-CD63 or HiBiT-HSP70 interaction with cytosolic or endosome bound LgBiT.

### CD63-EV cargo release into receiving cells can be measuredwith EVs carrying VSV-G

To confirm that the split NanoLuc assay was sufficiently sensitive to detect fusion events of EVs with membranes of receiving cells, we purified EVs from HEK293 cells co-expressing the VSV-G fusion protein with HiBiT-CD63. NTA analysis revealed that the total number of EVs was comparable between HEK293 cells expressing HiBiT-CD63 alone and those co-expressing HiBiT-CD63 with VSVG (Fig. 4a leftpanel), even if the size of EVs from VSV-G expressing cells was slightly increased (Fig. 4a middle panel).The orientation of CD63 in EVs purified from cells expressing both HiBiT-CD63 and VSV-G was not affected by VSV-G since it was not different from that of EVs containing only HiBiT-CD63 (Fig. 4a right panel).

WB analysis showed the presence of VSV-G in EVs purified from cells expressing both HiBiT-CD63 and VSV-G (Fig. 4b). We noticed that the amount of HiBiT-CD63 is significantly decreased in EVs secreted by cells also expressing VSV-G. Co-staining of EVs purified from cells expressing CD63 and VSV-G (Fig. 4c) showed that only a small fraction of the CD63-EVs carried VSV-G as already shown by Zhang et al.^15^.

**Fig. 5:**
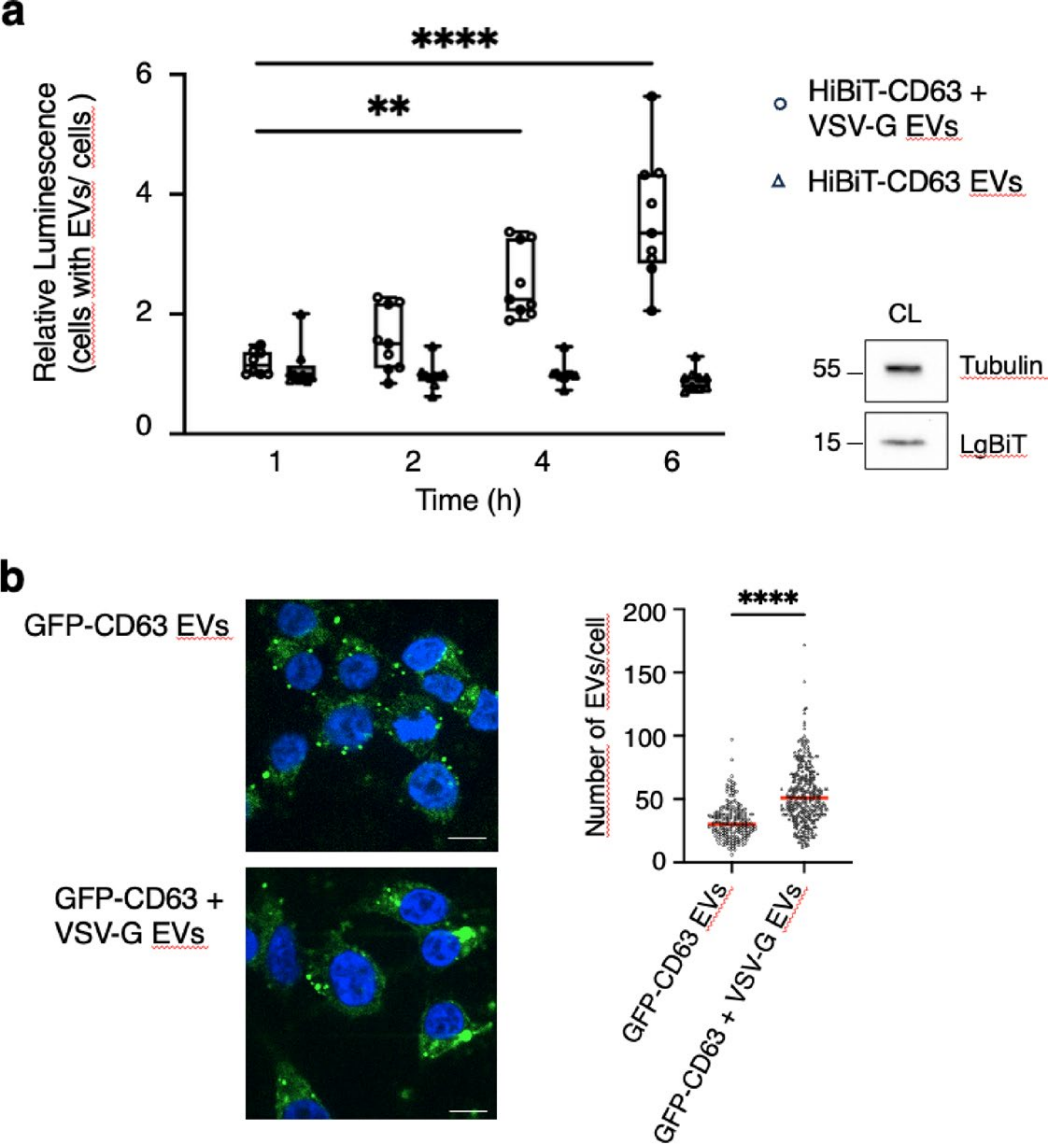
The presence of VSV-G in HiBiT CD63-EVs incubated on LgBiT-HEK293 cells strongly increases luminescence while mildly increasing cellular uptake. (**a**) EVs were incubated on LgBiT-cells and washed at the indicated times. DrkBiT was added 10 min before the Nano-Glo substrate and luminescence measured 20 min later. Median (q1; q3) of three wells from three independent experiments. Statistically significant differences were calculated using Kruskal-Walli’s test (**p < 0.01, ****p < 0.0001). Expression of LgBiT protein in receiving cells was revealed by anti-LgBiT antibody. (**b**) Cellular uptake of GFP-CD63 EVs is enhanced by VSV-G. GFP-CD63 EVs, with or without VSV-G, were incubated on HEK293 cells for 6 h. Internalized GFP-positive EVs were visualized in a single plane of z-stack confocal fluorescence images (Scale bare = 10 μm). Quantification of intracellular GFP signal was performed using full z-stack image analysis. Data represent the median number of internalized EVs per cell (*n* = 220 cells for GFP-CD63 EVs; *n* = 340 cells for GFP-CD63 + VSV-G EVs), pooled from three independent experiments. Statistical significance difference was calculated using the Kruskal–Walli’s test (****p < 0.0001).

However, this minimal amount of VSV-G was sufficient to induce fusion of CD63 EVs. Indeed, luminescence measured in LgBiT cells incubated with EVs purified from cells expressing both HiBiT-CD63 and VSV-G increased within 4 h, whereas no change was seen with EVs from cells expressing only HiBiT-CD63 (Fig. 5a), even with incubation up to 18 h (not shown). Next, we used GFP-CD63 EVs to test whether the presence of VSV-G in CD63 EVs could modify their uptake by recipient cells. We found that staining of HEK293 cells incubated for 6 h with GFP-CD63 EVs was increased by 1.7 times for EVs containing VSV-G. This indicates that, as expected^15^, VSV-G may not only facilitate fusion of EVs but also significantly increases their uptake by receiving cells (Fig. 5b).

Thus, the NanoBiT system is sensitive enough to monitor fusion of HiBiT-CD63 EVs to membranes of LgBiT expressing cells if EVs carry the fusion protein VSV-G.

## Discussion

Our study set out to employ the NanoBiT assay to assess whether extracellular vesicles (EVs) bearing HiBiT-CD63 could fuse with HEK293 recipient cells expressing LgBiT. The NanoBiT system, which relies on the high-affinity interaction between HiBiT and LgBiT (Kd ≈ 0.7 nM), was chosen for its remarkable sensitivity and specificity. This design ideally enables the detection of even rare fusion events by reconstituting an active luciferase enzyme upon membrane fusion. Our results demonstrate that the assay is indeed highly sensitive. When EVs containing HiBiT-CD63 were incubated with LgBiT-expressing cells, a robust and rapid increase in luminescence was evident when EVs were engineered to express the viral fusion protein VSV-G. This clear positive control confirms that the NanoBiT system is highly sensitive in reporting fusion events when they occur. The high potency of our system to report fusion is reinforced by the fact that VSV-G was present only in a low number of CD63 EVs consistent with the observation of Zhang et al. reporting that less than 5% of CD63-EVs incorporate VSV-G-GFP^15^. EVs containing HiBiT-CD63 but lacking VSV-G bound to and were possibly endocytosed by receiving cells but led to no increase in luminescence showing that in the absence of any fusogenic stimulus, the assay detects little to no membrane fusion between EVs and cell membranes.

Moreover, the use of the DrkBiT peptide to block signals arising from LgBiT leakage further attests to the assay’s specificity, ensuring that any luminescent readout is attributable solely to the fusion-mediated mixing of the HiBiT and LgBiT components. We also fused LgBiT to endosomal markers (RAB5 and RAB7) in order to concentrate it to endosomal membranes and thereby increasing the chance of interaction of HiBiT-CD63 with LgBiT. Here again, no increase of luminescence suggesting fusion of EVs to the limiting membrane of endosomes could be observed. Similarly, changing CD63 for HSP70 as a reporter of EV cargo release failed to generate a luminescence signal when incubated on LgBiT expressing cells. Similarly, Somiya et al. failed to detect luminescence in LgBiT expressing cells incubated with EVs containing HiBiT fused to a de novo designed protein (I3-01) that spontaneously forms a 60-mer self-assembled nanocage (EPN-01)^16^. Here again, a time increase in luminescence could only be detected when EPN-01 containing EVs also contained the fusion protein VSV-G^17^. Collectively, our findings together with those of the literature^8,9^ imply that CD63-EVs bind to cells and might primarily be internalized via endocytic pathways without significant membrane fusion—a scenario that could limit the functional delivery of their cargo. Noteworthy, is that EPN-01 containing EVs^17^ were shown to be secreted from the plasma membrane^16^ implying that the lack of fusion of EVs with recipient cells also concerns ectosomes.

Since we and others^8,9^ have demonstrated that endocytosis of CD63-EVs occurs, it seems surprising that these vesicles are not capable of fusing with the limiting membrane of endosomes unless they contain VSV-G. Indeed, a chemically tunable cell-based system allowed Perrin et al. to demonstrate that in endosomes, commitment to intraluminal vesicles (ILVs) carrying CD-63 is not a terminal event, and that a return pathway exists, allowing ‘‘back-fusion’’ of intraluminal membranes to the limiting membrane of multivesicular bodies^18^.However, they also demonstrate that only a pool of ILVs contributes to dynamics within MVBs allowing intraluminal proteins to return to the limiting membrane, whereas the other, more inert pool encompasses the bulk of secreted exosomes. This might explain why CD63-EVs are not capable of fusing with endosome limiting membranes of receiving cells.

It is important to note that our conclusions are primarily based on experiments using HEK293 cells as both donor and recipient cells. De Jong et al.^19^ reported with a CRISPR-Cas9-based reporter system that the efficiency of functional RNA transfer via EVs depends on the specific combination of donor and recipient cell types. Nevertheless, we observed similarly negative results when testing HiBiT-CD63 EVs on other recipient cell lines, including MCF-7 and HeLa cells, which have previously been reported to endocytose HEK-derived CD63 EVs^4,20^.

In conclusion, our study demonstrates that the NanoBiT assay is a sensitive and specific method for detecting EV membrane fusion. The absence of detectable fusion by unmodified EVs, in contrast to the robust signal observed with VSV-G–modified EVs, suggests that CD63-positive EVs may not efficiently enter and fuse with recipient cell membranes under the conditions tested. Importantly, several studies have suggested that overexpressed CD63 or CD63-GFP labels only about half of the total extracellular vesicle (EV) population, highlighting the need for further investigation into the fusion capabilities of natural EVs. However, alongside growing evidence pointing to the limited ability of EVs to deliver functional cargo to recipient cells unless modified by fusion proteins, our results call into question the widely held view that EVs are key mediators of intercellular communication through cargo transfer.

## Supplementary Information

Below is the link to the electronic supplementary material.

Supplementary Material 1

## Data Availability

The datasets generated during the current study are available from the corresponding author on reasonable request.
