# Supplementary figures and images for: Lack of evidence for cargo release of CD63-EVs into recipient cells
Source: Sci Rep. 2026 Mar 26;16:15164. doi: 10.1038/s41598-026-45021-2 (PMC13171887; doi:10.1038/s41598-026-45021-2)

Fig.1a

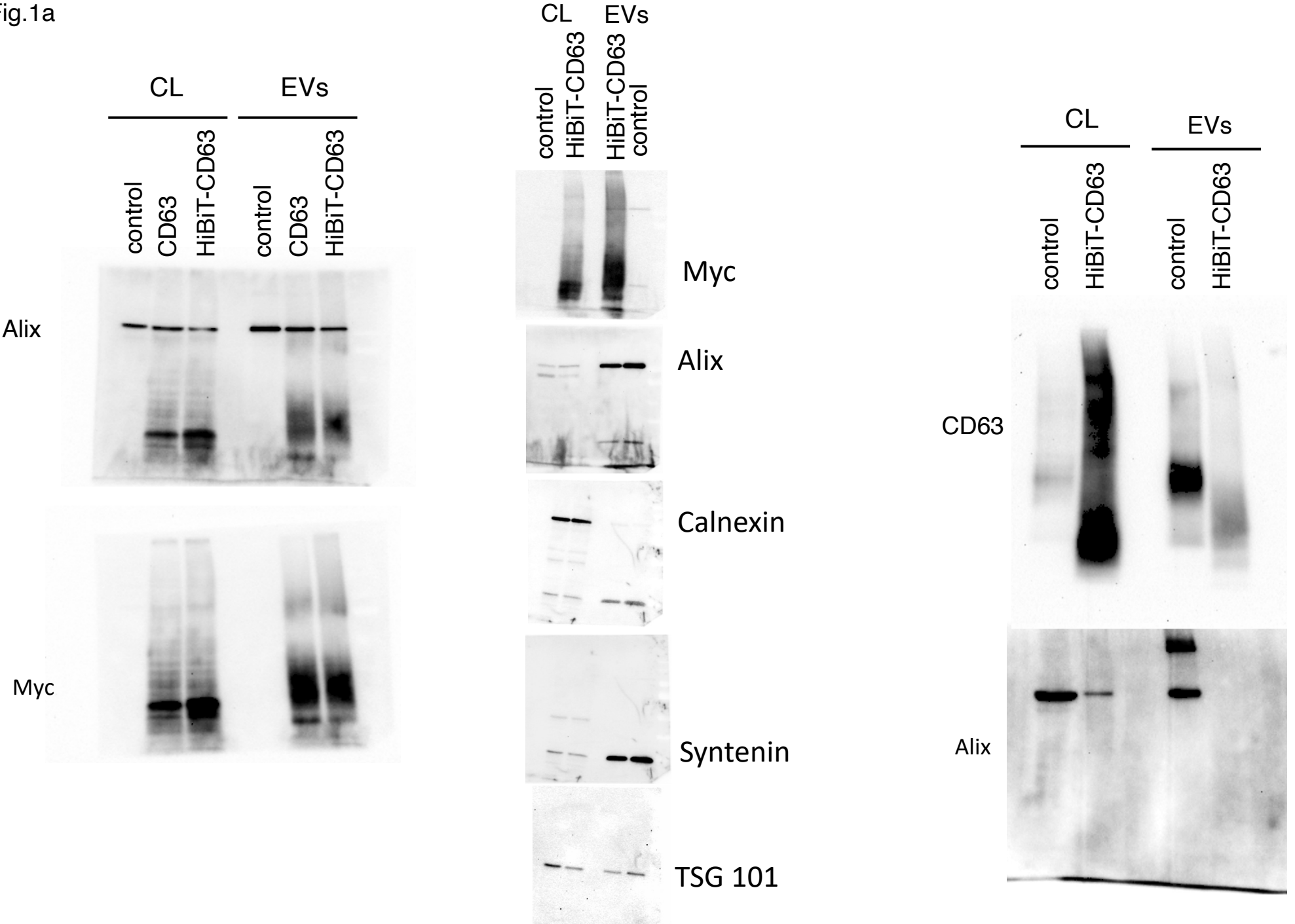

Fig. 3c

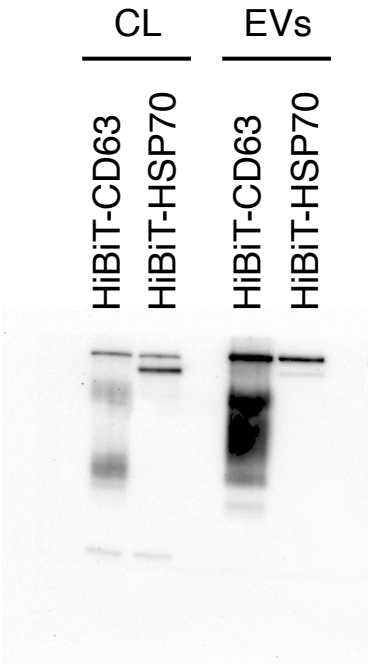

Fig.4b

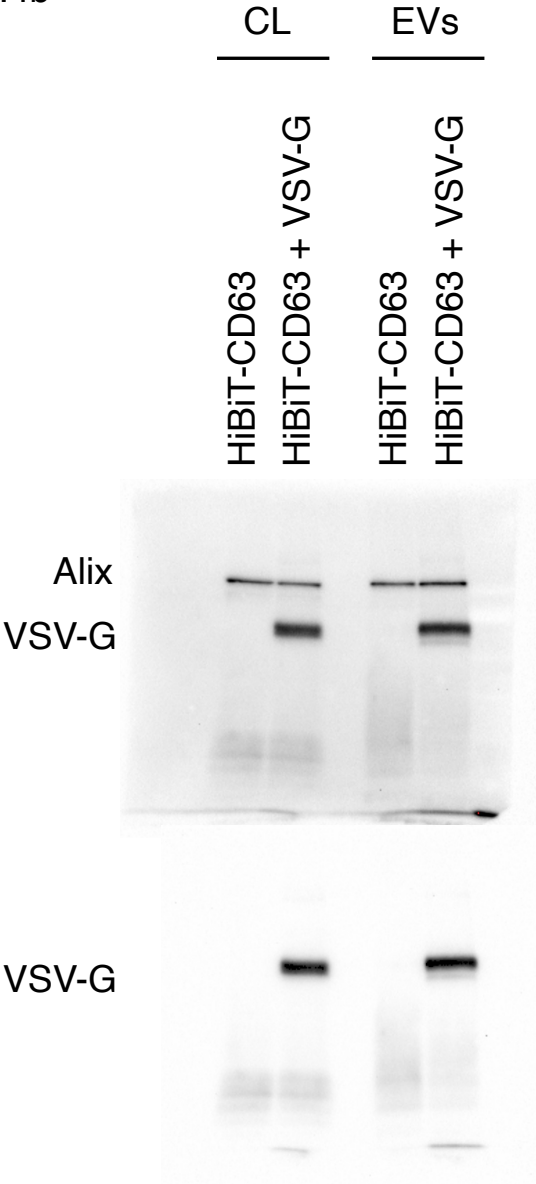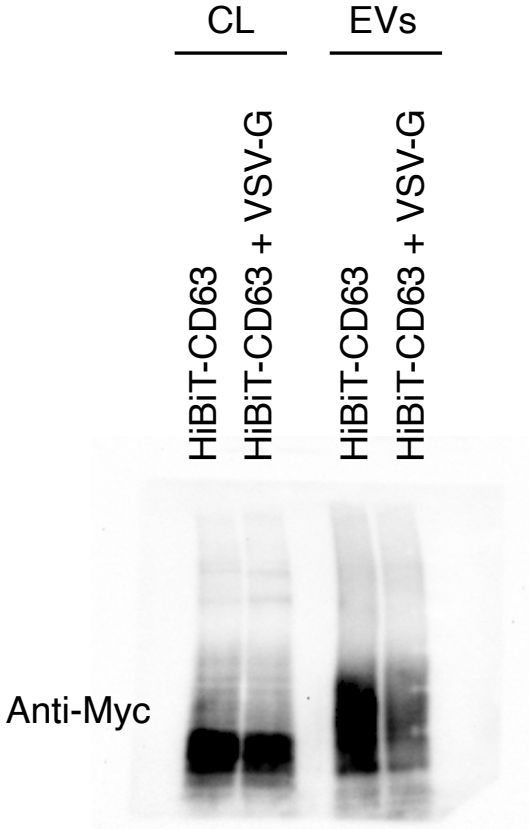

Fig 2a

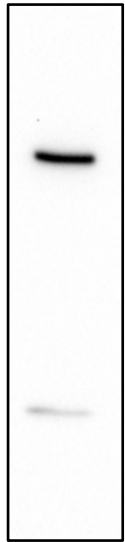

Fig 3a

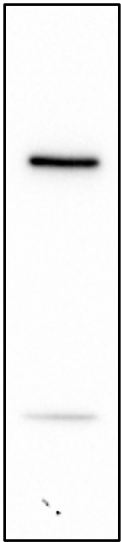

Fig 5a

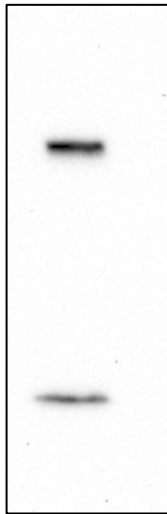

Tubulin

LgBiT

Fig 3b

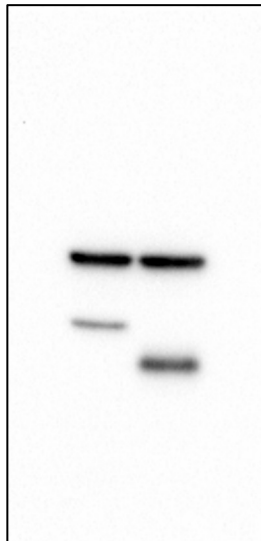

Tubulin

LgBiT RAB5

LgBiT RAB7

Supplement: Supplementary file 1 — Supplementary Material 1 [file 41598_2026_45021_MOESM1_ESM.pdf]
